# What’s That (Blue) Spot on my MRI? Multimodal Neuroimaging of the Locus Coeruleus in Neurodegenerative Disease

**DOI:** 10.3389/fnins.2020.583421

**Published:** 2020-10-06

**Authors:** Michael Kelberman, Shella Keilholz, David Weinshenker

**Affiliations:** ^1^Department of Human Genetics, Emory University, Atlanta, GA, United States; ^2^The Wallace H. Coulter Department of Biomedical Engineering, Georgia Institute of Technology, Emory University, Atlanta, GA, United States

**Keywords:** locus coeruleus, neurodegeneration, Alzheimer’s disease, Parkinson’s disease, magnetic resonance imaging, positron emission tomography, animal models

## Abstract

The locus coeruleus (LC) has long been underappreciated for its role in the pathophysiology of Alzheimer’s disease (AD), Parkinson’s disease (PD), and other neurodegenerative disorders. While AD and PD are distinct in clinical presentation, both are characterized by prodromal protein aggregation in the LC, late-stage degeneration of the LC, and comorbid conditions indicative of LC dysfunction. Many of these early studies were limited to post-mortem histological techniques due to the LC’s small size and location deep in the brainstem. Thus, there is a growing interest in utilizing *in vivo* imaging of the LC as a predictor of preclinical neurodegenerative processes and biomarker of disease progression. Simultaneously, neuroimaging in animal models of neurodegenerative disease holds promise for identifying early alterations to LC circuits, but has thus far been underutilized. While still in its infancy, a handful of studies have reported effects of single gene mutations and pathology on LC function in disease using various neuroimaging techniques. Furthermore, combining imaging and optogenetics or chemogenetics allows for interrogation of network connectivity in response to changes in LC activity. The purpose of this article is twofold: (1) to review what magnetic resonance imaging (MRI) and positron emission tomography (PET) have revealed about LC dysfunction in neurodegenerative disease and its potential as a biomarker in humans, and (2) to explore how animal models can be used to test hypotheses derived from clinical data and establish a mechanistic framework to inform LC-focused therapeutic interventions to alleviate symptoms and impede disease progression.

## Introduction

The locus coeruleus (LC) is a compact brainstem nucleus consisting of approximately 30,000–50,000 neurons in adult humans (Mouton et al., 1994; Aston-Jones and Cohen, 2005b). Although small, the LC sends dense afferent projections throughout the brain, supplying its target regions with norepinephrine (NE) and other co-transmitters to influence cognition, attention, stress response, and arousal (Aston-Jones and Cohen, 2005b; Benarroch, 2018). In addition to its many functions, the LC is of high clinical relevance for neurodegenerative diseases, particularly Alzheimer’s disease (AD) and Parkinson’s disease (PD). Changes to LC function in AD, PD, and a host of other neurodegenerative diseases have been extensively described elsewhere (Weinshenker, 2018; Betts et al., 2019b; Janitzky, 2020), but we will highlight a few key studies. Despite presenting with unique primary symptoms and pathologies, both AD and PD share the common feature of LC impairment. The LC exhibits prodromal accumulation of hyperphosphorylated of tau in AD (Braak et al., 2011; Pletnikova et al., 2018) and α-synuclein aggregation in PD (Tredici et al., 2013), often well before other areas that are canonically associated with these disorders such as the hippocampus, cortex (AD), and basal ganglia (PD). Interestingly, while protein aggregates are thought to trigger neural degeneration in other brain regions, the LC appears to be spared from frank cell death until mid to late stages of disease, when up to 80% of its cell bodies are lost (German et al., 1992; Busch et al., 1997; Zarow et al., 2003; Theofilas et al., 2017). Furthermore, AD and PD share many comorbidities associated with LC dysfunction, such as exaggerated stress response and anxiety (Teri et al., 1999; Pietrzak et al., 2015; Schrag and Taddei, 2017), attentional deficits (Rizzo et al., 2000; Berardi et al., 2005; Dujardin et al., 2013), fatigue and reduced arousal (Hamann et al., 2000; Kelberman and Vazey, 2016) and sleep disturbances (Yesavage et al., 2004; Ju et al., 2013) that appear time-locked with development of LC pathology (Busch et al., 1997; Grinberg et al., 2010; Ehrenberg et al., 2018).

Due to its small size and location deep in the brainstem apposed to 4th ventricle, initial study of LC dysfunction in humans was largely limited to post-mortem histological approaches. However, with advances in various neuroimaging techniques, *in vivo* structural and functional imaging of the LC, NE, and its related neuropathologies are now invaluable experimental tools for analysis of noradrenergic dysfunction in neurodegenerative disease. While many of these approaches have been adopted for clinical studies, LC imaging has rarely been explored in animal models, which represent our best opportunity to parse out single gene risk variants and pathology-specific contributions to LC dysfunction in neurodegenerative disease. Furthermore, the use of animal models allows for longitudinal imaging that is often constrained by time, cost, and compliance in human populations. Having already briefly summarized major changes to the LC-NE system in neurodegenerative diseases, we will first focus on how *in vivo* imaging of the LC in humans has enriched our understanding of its dysfunction in neurodegenerative processes. Then, we will highlight the potential that imaging in animal models offers for studying abnormalities in the LC itself, as well as its impact on brain-wide networks during disease progression.

## Human Studies

Developing *in vivo* imaging methods targeting the LC is of great interest given prior evidence of its involvement in various neurodegenerative disorders. Imaging approaches can be separated based on the particular aspect of LC health that they interrogate, including LC volume, axonal integrity, changes in functional connectivity at-rest or during task engagement, pathological load, and its relation to any previously mentioned factor. The LC is difficult to reliably segment from other nuclei due to its small size and location deep within the brainstem, making the application of traditional structural and functional imaging techniques challenging. A major breakthrough in imaging methods was the discovery of neuromelanin-sensitive magnetic resonance imaging (MRI) sequences, which have allowed accurate segmentation of the LC in MRI and estimations of LC integrity. As a complement to MRI, diffusion tensor imaging (DTI) can be used to assess axonal health. Positron emission tomography (PET) can also be used to proxy neuron integrity by imaging the NE transporter (NET), which is distributed on LC dendrites, somas, axons, and terminals. PET has additional utility for assessing pathological load, particularly with regards to the LC, which develops various neuropathological hallmarks of disease, even in prodromal phases. We will cover each of these methods in relation to the LC and neurodegenerative disease in the following sections, highlighting progress that has been made in development and application, study outcomes, and data interpretation. We also underscore limitations in deployment and analysis of results for each method, as well as comment on how these imaging modalities can be applied in future studies.

### Structural MRI

**FIGURE 1:**
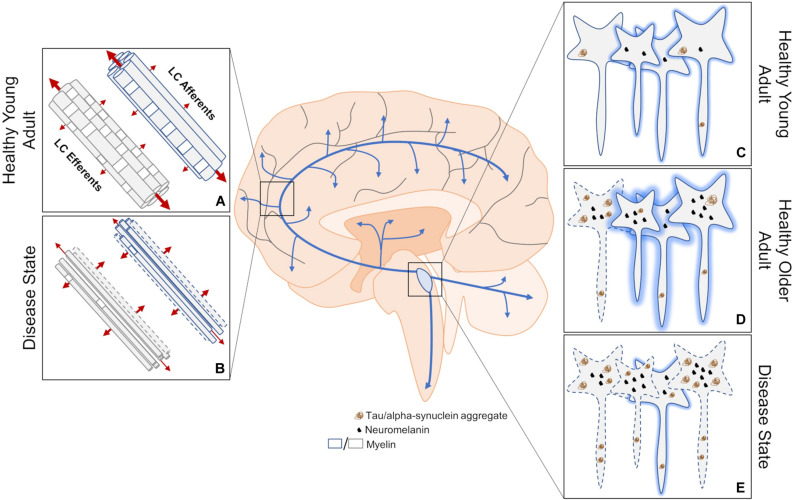
An overview of structural MRI approaches to investigating changes in LC dysfunction in neurodegenerative disorders. **(A)** DTI measures three-dimensional diffusion of water molecules (red arrows where thickness of the arrow represents weight of diffusion) to get readouts of axonal health. Water molecules tend to diffuse along, rather than across, healthy myelinated axons. **(B)** This structural imaging method has been used to determine changes in innervation, myelination, and axon size between the LC and downstream brain regions in disease states, but cannot distinguish between deficits in efferent or afferent connections. **(C)** Contrast thought to arise from neuromelanin, high water proton density, and/or other mechanisms allow the LC to be visualized using structural MRI. **(D)** LC contrast appears to peak in adulthood, which is correlated with neuromelanin levels. At this stage, some hyperphosphorylated tau may be apparent. **(E)** A decrease in LC contrast is thought to represent compromised LC integrity during disease states, but has yet to be attributed to cell or dendritic field loss. Neurodegeneration may result from the aggregation of hyperphosphorylated tau. Dashed lines indicate degenerating neurons and shading represents relative contrast from each neuron. Blue arrows represent LC-NE release sites throughout the brain and spinal cord.

Structural MRI can take many forms including volumetric analysis, DTI, and contrast that is specific to select nuclei like the LC (Figure 1). MRI scans are minimally invasive, widely available, and offer superior spatial resolution compared to other techniques such as PET, making it an ideal method to study the LC. Structural analyses have been widely adopted in the clinic to assist with diagnosis of neurodegenerative disorders and assess whether certain treatments can ameliorate brain atrophy. Structural MRI can also be used alone or in concert with other imaging techniques to identify new regions of interest and correlate atrophy in these regions with neuropathology. The major development in *in vivo* LC imaging was the discovery that scanning protocols sensitive to neuromelanin could be used to visualize the LC (Figures 1C,D) (Sasaki et al., 2006). Neuromelanin, a byproduct of catecholamine synthesis and metabolism, is a dark pigment that accumulates in the LC and other catecholaminergic nuclei (most notably the substantia nigra pars compacta) with age. Accumulating evidence suggests that multiple sources are responsible for LC hyperintensities, including magnetization transfer and T1 shortening (Sasaki et al., 2006; Keren et al., 2009; Betts et al., 2017; Priovoulos et al., 2018). The use of LC contrast as an early indicator of LC-NE dysfunction in neurodegenerative diseases has been recently reviewed (Betts et al., 2019b), and we direct readers to this article for an overview of common LC imaging parameters in humans. We will briefly summarize the major studies highlighted by this previous review, and then add additional commentary on non-LC contrast measurements of structure in neurodegenerative disease.

LC contrast is lower in AD and PD (Figure 1E) (Sasaki et al., 2006; Takahashi et al., 2015; Dordevic et al., 2017; Wang et al., 2018; Betts et al., 2019a; Olivieri et al., 2019). In AD, lower LC contrast is localized to the rostral and middle thirds of the nucleus (Betts et al., 2019a), which is in agreement with previous postmortem reports indicating patterned LC volume loss in AD but not PD (German et al., 1992; Theofilas et al., 2017). Deterioration of the rostral and middle portions of the LC generally affect hippocampal and cortical innervation (Loughlin et al., 1986; Sara and Bouret, 2012), which is consistent with the progressive symptomology of AD. Some imaging parameters can lead to biases in segmentation of the LC along its rostrocaudal extent (Liu K. Y. et al., 2017; Liu et al., 2019), such that the caudal and middle segments encompass portions of the middle and rostral LC, respectively. However, Betts et al. (2019a) used isotropic acquisition parameters, which may be more reliable in capturing and segmenting the rostrocaudal extent of the LC (Betts et al., 2019b). Although no differences in LC contrast are noticeable between mild-cognitively impaired and AD patients (Takahashi et al., 2015), LC contrast may be better than substantia nigra contrast at discriminating PD patients from healthy controls (Ohtsuka et al., 2013). Differences in LC contrast can also be detected between similar disorders like PD, multiple system atrophy, and progressive supranuclear palsy (Matsuura et al., 2013; Ohtsuka et al., 2014). REM Sleep Behavior Disorder (RBD) is among the earliest and most common prodromal PD syndrome, and PD patients with concomitant RBD show substantial reduction in LC contrast compared to those without RBD (Sommerauer et al., 2018a). Indeed, even patients with depression (Shibata et al., 2007, 2008) or RBD (Ehrminger et al., 2016; Knudsen et al., 2018) in the absence of a clinical PD diagnosis present with lower LC contrast, suggesting that changes in LC contrast could represent a prodromal biomarker of neurodegenerative disease. Longitudinal work is needed to determine whether changes in LC contrast paralleled by early behavioral biomarkers predict future development of neurodegenerative disease. In healthy older adults, higher LC contrast is positively associated with cortical thickness (Bachman et al., 2020), better memory performance (Hämmerer et al., 2018; Dahl et al., 2019), and preserved cognition (Liu et al., 2020). Early evidence suggests that even in subjects with typical AD presentation, high LC contrast is associated with better memory performance (Olivieri et al., 2019). These studies are consistent with reports that postmortem LC integrity appears to encode neural reserve and cognitive function during healthy aging (Robertson, 2013; Wilson et al., 2013), and LC contrast does correlate with known measures of neural reserve (Clewett et al., 2016). It should be noted that using LC contrast to estimate neuronal integrity could be confounded by age-related increases in LC neuromelanin content. Studies of healthy individuals suggest that neuromelanin accumulates until approximately age 60, and then plateaus or declines (Mann and Yates, 1974; Manaye et al., 1995; Zucca et al., 2006). Additionally, early histological evidence suggested that LC degeneration occurs during the course of normal aging (Vijayashankar and Brody, 1979; Manaye et al., 1995), but these findings have also been refuted (Mouton et al., 1994; Theofilas et al., 2017). Thus, whether patterns of LC contrast over the course of normal aging represents cessation of neuromelanin production, changes in neuron morphology, and/or are indicative of cell or dendrite loss is a hotly contested subject (Clewett et al., 2016; Liu et al., 2019; Morris et al., 2020). While volumetric analysis of the LC also suffers from issues in segmentation (Liu K. Y. et al., 2017; Betts et al., 2019b), utilizing isotropic acquisition parameters and combining it with postmortem stereological counts may help disentangle the changes to LC structure over the course of disease.

DTI is a MRI technique that is used to estimate axonal health by measuring three-dimensional diffusion of water molecules (Figures 1A,B). Lower fractional anisotropy is commonly reported in areas that are known to degenerate over the course of various diseases (Nilsson et al., 2007; Kitamura et al., 2008; Kantarci et al., 2010; Nave et al., 2011), and is thought to reflect reduced axonal integrity but is otherwise non-specific. Radial and axial diffusivity are associated with myelination and axonal degeneration, respectively (Alexander et al., 2007). A majority of LC fibers are thought to be unmyelinated, with the exception of those innervating the neocortex (Aston-Jones et al., 1985). However, increases in both radial and axial diffusivity are noted in fibers connecting the right hypothalamus and right LC in multiple sclerosis patients without self-reported fatigue (Hanken et al., 2016), suggesting outright axonal degeneration but also the presence of more widespread LC axon myelination than previously reported. An alternative explanation is that axonal degeneration and demyelination are specific to reciprocally projecting fibers emanating from the hypothalamus, which cannot be ruled out by DTI. In progressive supranuclear palsy, lower fractional anisotropy of the LC and other regions was noted compared to both PD patients and healthy controls (Pyatigorskaya et al., 2020). Fractional anisotropy of the LC did not differ between PD patients and healthy controls, which could be explained by lack of clinical staging. Measures of mean, radial, and axial diffusivity would help clarify precise changes to LC axons over the course of disease. In healthy participants, aging appears to increase fractional anisotropy and lowers both mean and radial diffusivity of the LC (Langley et al., 2020). The authors propose decreased LC axon diameter underlie these results, but follow up studies should directly test this hypothesis. These three measures of LC integrity were correlated with Rey Auditory Verbal Learning Test scores, but only in older adults, which is again consistent with the association between LC integrity and neural reserve (Robertson, 2013; Wilson et al., 2013). A non-significant decrease in fractional anisotropy between the LC and parahippocampal gyrus has also been reported in mild cognitively impaired individuals compared to cognitively normal controls (Jacobs et al., 2015). Caution must be taken when interpreting DTI results of LC structure due to the inherently low signal-to-noise ratio, and susceptibility to partial volume effects and varying fiber orientations that are exaggerated in small nuclei (Soares et al., 2013; Betts et al., 2019b). Newer techniques like diffusion kurtosis imaging (Jensen et al., 2005), neurite orientation dispersion and density imaging (Zhang et al., 2012), or field strengths greater than 7T may help, but increases in acquisition time could introduce motion artifacts that are also problematic when imaging small nuclei. Thus, further methodological improvements, and perhaps specialized imaging sequences, could be developed for future imaging of LC fibers.

### Functional MRI

Functional MRI (fMRI) was developed to assess patterns of brain activation by indirectly measuring neuronal activation through changes in blood flow. Because functional scans require the acquisition of anatomical scans to coregister brain regions for functional analyses, data for functional and anatomical scans are often acquired within the same session. Scans are then typically spatially normalized to brain atlases and the LC is localized using structural maps (Liu K. Y. et al., 2017), such as the popular one published by Keren et al. (2009), which presents a probabilistic location of the LC in Montreal Neurological Institute coordinate space. Nonetheless, special consideration should be given to factors such as accuracy of coregistration, potential partial volume effects, and tradeoffs between smaller voxel size and adequate signal-to-noise due to the LC’s location and small size (Düzel et al., 2015; Liu K. Y. et al., 2017; Betts et al., 2019b). fMRI can also be performed at rest or during a specific task to detect abnormalities in brain connectivity during different states. However, functional imaging of the LC is complicated by the fact that the LC can fire in two distinct patterns (tonic and phasic) thought to give rise to exclusive brain and behavioral states (Foote et al., 1980; Aston-Jones and Bloom, 1981; Akaike, 1982; Grant et al., 1988; Sara and Segal, 1991; Aston-Jones et al., 1999; Aston-Jones and Cohen, 2005a), which may induce specific patterns of functional connectivity. Moreover, the LC is surrounded by a pericoeruleur dendritic zone rich in GABAergic neurons (Swanson, 1976; Aston-Jones et al., 2004; Breton-Provencher and Sur, 2019). Parsing out the functional connectivity contributions of these LC-specific factors is thus of the utmost importance (Düzel et al., 2015). Finally, while the LC has historically been considered a homogenous nucleus due to its near universal projection and seemingly synchronous firing patterns, more recent evidence indicates modularity (Chandler et al., 2014; Uematsu et al., 2017; Totah et al., 2018; Noei et al., 2020), which should be taken into account when analyzing the LC as a region of interest using fMRI. Given these considerations, only recently have studies begun to investigate direct connectivity of the LC, and have treated it as a single nucleus, as opposed to segmenting it along the rostrocaudal axis. Although current results at 3 and 7T are largely congruent (Zhang et al., 2016; Jacobs et al., 2018; Liebe et al., 2020), these preliminary experiments should be replicated and expanded using tasks known to evoke LC activity, at multiple points during disease states, and at higher resolution.

**FIGURE 2:**
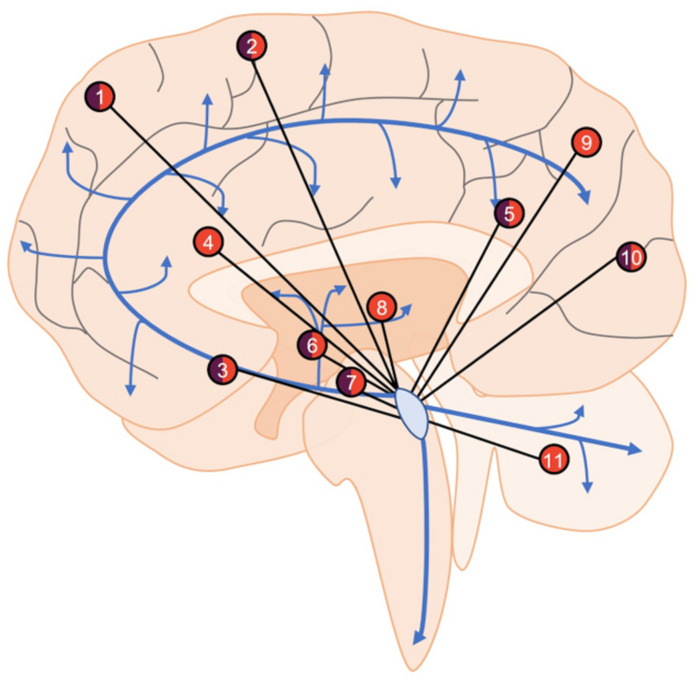
An overview of LC functional connectivity obtained using fMRI. Areas with positive (orange), negative (purple), or mixed (both) functional connectivity with the LC include frontal **(1)** and sensorimotor **(2)** cortical regions, nucleus basalis of Meynert **(3)**, anterior and posterior cingulate cortices **(4, 5)**, caudate/putamen **(6)** ventral tegmental area **(7)**, thalamus **(8)**, parietal cortex **(9)**, occipital cortex **(10)**, and cerebellum **(11)**. Areas showing both positive and negative functional connectivity are the result of specific subregions of interest (i.e., in the frontal cortex, the LC is positively connected with superior frontal gyrus but negatively connected with frontopolar regions), changes in connectivity across aging (i.e., nucleus basalis of Meynert), or disparate findings between studies (i.e., occipital cortex). Blue arrows represent LC-NE release sites throughout the brain and spinal cord.

In healthy individuals at rest, the LC shows both negative and positive functional connectivity to a variety of brain regions such as the cingulate cortex, thalamus, cerebellum, and various frontal, parietal, and temporal regions (Figure 2) (Bär et al., 2016; Zhang et al., 2016; Jacobs et al., 2018; Liebe et al., 2020). These studies are highly consistent, but slight differences are apparent and are likely due to different imaging parameters, preprocessing protocols, and analyses. There are also notable changes in LC connectivity during the course of aging; functional connectivity with the cerebellum and fronto-parietal cortices appears to increase over time (Zhang et al., 2016). In healthy adults with a parental history of AD, functional connectivity between the LC and cerebellar cortex is decreased (Del Cerro et al., 2020). These at-risk individuals were slightly older than those from studies involving typical individuals, suggesting that the deficits could be due to either a loss of functional connectivity or a failure to increase during aging. LC connectivity with the ventral tegmental area and salience networks decreases with age (Jacobs et al., 2018; Lee et al., 2020), while various forebrain regions and the nucleus basalis of Meynert demonstrate non-linear functional connectivity variation with the LC during aging (Jacobs et al., 2018). These diverse connectivity patterns across aging are also associated with cognitive outcomes. Higher LC-nucleus basalis of Meynert or ventral tegmental area connectivity is associated with poorer memory in those over 40 (Jacobs et al., 2018), whereas stronger connectivity with the parahippocampal gyrus is associated with better memory performance (Jacobs et al., 2015). Additionally, in individuals with mild-cognitive impairment, LC-parahippocampal gyrus connectivity is disrupted, but still correlates with memory performance specifically in high functioning individuals. Other methods such as Granger Causality can identify directional functional interactions, whereby activity in one region precedes activity in another. Using this method, one study identified the LC as the node of functional connectivity deficits in AD patients (Zhao et al., 2017). These deficits were shown to culminate in the orbitofrontal cortex, taking multiple routes through the working memory, emotional memory, and language system circuits. Serra et al. (2018) failed to identify changes in LC functional connectivity in AD, but the authors concluded that LC visualization was outside the resolution capabilities of their MRI data. Moreover, when the same authors used less stringent statistical criteria, they observed changes in LC connectivity. Together, these early studies indicate certain LC functional connectivity patterns could also represent a proxy of neural reserve beyond previously described structural integrity.

Because all published studies have tested healthy subjects, those at-risk for AD, or individuals with a bona fide AD diagnosis, investigation of changes to LC functional connectivity in other neurodegenerative disorders is warranted. Notably, PD is diagnosed by motor dysfunction that has largely been localized to the basal ganglia, with LC involvement mostly disregarded due to the sparse noradrenergic input to this region (Berridge and Waterhouse, 2003). However, resting-state fMRI studies have provided evidence of noradrenergic contributions to the striatum (Zhang et al., 2016; Lee et al., 2020; Liebe et al., 2020), and it was recently reported that the LC directly innervates the caudate and putamen in mice (Zerbi et al., 2019). Stimulation of LC-striatal fibers in mice elicited NE release and increased overall functional connectivity. Further application of fMRI could uncover a direct noradrenergic influence on PD pathophysiology via a novel LC-basal ganglia circuit.

Previously discussed fMRI studies have been limited to correlating measures of LC functional connectivity and tasks that have been performed outside the scanner. However, it is possible to measure patterns of LC connectivity while performing NE-sensitive tasks. Task-relevant information appears to evoke phasic LC discharge, whereas tonic LC activity promotes task disengagement and behavioral flexibility (Aston-Jones et al., 1999; Aston-Jones and Cohen, 2005b). During a Stroop Color-Word task, incongruence between word and color elicited increased blood oxygen level dependent (BOLD) signals bilaterally in the LC compared to congruent conditions (Köhler et al., 2016). The LC was shown to be functionally connected with the prefrontal, motor, sensory, cingulate, and cerebellar cortices during task engagement. Furthermore, interference scores (reaction time to incongruent minus congruent conditions) positively correlated with LC BOLD activation. In a separate study, subjects were administered modafinil, a weak NET inhibitor, to decrease tonic firing and promote task-evoked phasic LC activity (Minzenberg et al., 2008). With modafinil on board, LC BOLD signal decreased at rest likely reflecting lower tonic activity, but increases between the LC and prefrontal cortex were noted during task engagement, which is indicative of higher phasic discharge. Collectively, these studies suggest that phasic LC activity or increases in signal-to-noise ratio (phasic versus tonic firing) may selectively activate task-relevant brain networks. Although evidence of task-based changes to LC connectivity in disease states for comparison is sparse, analysis of pre-existing datasets may be possible if they were collected under conditions conducive to LC segmentation (Liu et al., 2019). Further task refinement that makes them more specific to LC activity will be required moving forward, and could be aided by simultaneous pupillometry, as LC activation correlates with pupil dilation (see Box 1). In particular, tasks incorporating emotional events could be used to assess LC activity-related deficits in disease, especially during encoding and retrieval (Sterpenich et al., 2006; Jacobs et al., 2020).

**Box 1. Pupillometry as a window into LC activity and dysfunction. Many functional imaging studies take advantage of the fact that pupillary dilations parallel increases in LC activity, allowing for rapid and easy estimations of LC activity during tasks. LC activity-evoked pupillary responses are particularly noticeable as cognitive demand increases (Alnaes et al., 2014; Murphy et al., 2014; Joshi et al., 2016). Older adults demonstrate delayed and lower pupillary dilation compared to younger adults that can be partially restored by engaging phasic LC activity (Hämmerer et al., 2017, 2018; He et al., 2020). Individuals with mild cognitive impairment or high AD risk show increased task-evoked pupillary dilation, which is indicative of exaggerated LC activity under cognitive load (Granholm et al., 2017; Kremen et al., 2019). Regardless of cognitive status, higher tonic LC activity measured by low frequency BOLD variance appears to preclude task-related phasic activity-induced pupillary dilation (Elman et al., 2017), which is in line with unmasking of task-related increases in LC-prefrontal cortex connectivity due to phasic activity elicited by modafinil (Minzenberg et al., 2008). Together, these studies suggest that abnormal signal-to-noise ratio of the LC may be an early marker of disease that can be cheaply and reliably monitored through task-evoked pupil dilation. In support of using pupillary responses as a marker of LC-related cognitive dysfunction, non-demented PD patients show normal pupillary responses (Kahya et al., 2018). There is also evidence of disease-specific pupillary deficits, as PD patients show lower pupillary dilation in response to high cognitive demand tasks involving motor preparation (Wang et al., 2016). Caution should be taken when comparing these results because AD cases were preclinical, while the PD cases were symptomatic, and the use of different tasks could influence pupillary response. Pupillometry is also possible in primates and rodents, and simultaneous pupillometry with fMRI has recently been described (Pais-Roldán et al., 2020). Animal studies have been important for demonstrating that direct LC stimulation and inhibition can elicit pupil dilation and constriction (Liu Y. et al., 2017; Zerbi et al., 2019; Hayat et al., 2020), respectively. Animal models would also be useful for comparing the effects of phasic and tonic firing patterns pupil dilation, which could further refine our understanding of LC activity in human studies during task engagement.**

### Positron Emission Tomography

**FIGURE 3:**
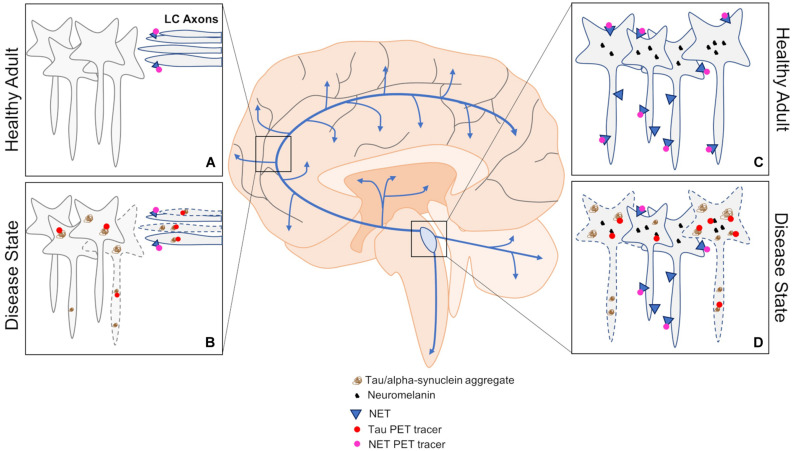
An overview of PET approaches to investigating changes in LC dysfunction in neurodegenerative disorders. **(A,B)** Tau and NET PET can be used to assess pathological load and LC fiber integrity by imaging downstream brain regions in healthy and disease states. **(C,D)** Imaging the LC using tau and NET PET can also be informative of pathological load and cell body integrity in healthy and disease states. Blue arrows represent LC-NE release sites throughout the brain and spinal cord.

PET is a functional imaging procedure that utilizes radioligands to assess changes in metabolism, neurotransmitter levels, and other markers of interest. Compared to MRI, PET has poorer spatial resolution, higher costs, and is more invasive, but has the distinct advantage of being able to measure LC dysfunction in terminal regions and determine pathological load associated with neurodegenerative diseases, depending on the tracer used (Figure 3). NET, which is localized to LC axons, dendrites, and cell bodies, can be assessed with PET in regions innervated by the LC, offering another method to assess LC structure. Therefore, with respect to the LC, PET sits at a unique crossroads between structural and functional imaging. Outcomes from PET studies can also be correlated to, or used as a predictor of, various outcome measures including LC integrity and functional connectivity. Unfortunately, PET has suffered from tracer development issues, particularly with respect to assessing pathological load within the LC due to low resolution and off-target binding. Therefore, use of LC PET imaging as a diagnostic tool and biomarker of neurodegenerative disease is limited for now, but has the potential for future application with the advent of new generations of tracers.

Approximately 16 years ago, the first amyloid PET study was performed in humans using Pittsburgh Compound-B (Klunk et al., 2004), and it continues to be widely employed today. However, in AD, the LC appears to be selectively vulnerable to developing tau pathology, and is relatively spared from plaque deposition expect in severe cases or during late stages of disease. Tau tracers have also been developed and broadly fall into three categories (Saint-Aubert et al., 2017), but have suffered from problems with off-target binding and lack of overlap with validated antibodies (Sander et al., 2016; Marquié et al., 2017; Vermeiren et al., 2018). Some of the issues with development likely arise from the necessity of tau tracers to penetrate cell membranes, since misfolded tau forms intracellular inclusions, and the numerous isoforms of misfolded tau that are often disease specific. Nevertheless, PET studies confirm previous reports of tau as a better predictor of cognitive decline and cell death in AD compared to amyloid (Cho et al., 2016; Kotzbauer et al., 2017; Gordon et al., 2018; Pereira et al., 2020). Newer tracers are being developed, and those such as 18F-RO-948 demonstrate an inverse relationship with cortical thickness (Spotorno et al., 2020), which is in agreement with older tracers (Ossenkoppele et al., 2019). Moreover, retention of 18F-RO-948 appears to be associated with cortical iron levels, which are also inversely correlated with cortical thickness. This is significant in the context of the LC because neuromelanin binds iron and other heavy metals. However, iron accumulation in the LC appears modest in normal aging (Zucca et al., 2006), and separate populations of LC neurons appear to accumulate tau and other heavy metals in AD (Pamphlett and Kum Jew, 2015). Other reports indicate highly variable concentrations of heavy metals in the LC (Pamphlett et al., 2020), and increasing concentrations of inorganic mercury during aging and in those with sporadic amyotrophic lateral sclerosis (Pamphlett and Kum Jew, 2013; Pamphlett et al., 2018). Therefore, a more comprehensive investigation of the links between neuromelanin, heavy metal content, and LC susceptibility to developing neuropathology or degeneration is warranted. Interestingly, although the LC develops pathology decades prior to clinical onset, it is relatively spared from degeneration until clinical presentation, which suggests that neuromelanin accumulation may work in concert with neuropathology to trigger neurodegeneration (Weinshenker, 2018; Vila, 2019). Specifically, neuromelanin sequesters catecholamine metabolites, and the noradrenergic-specific metabolite DOPEGAL can trigger tau cleavage, hyperphosphorylation, and LC degeneration (Weinshenker, 2018; Kang et al., 2020). Thus, accumulation and subsequent breakdown of neuromelanin granules during aging could potentially release large quantities of DOPEGAL, triggering tau pathology. Correlating LC tau PET and neuromelanin-sensitive contrast could shed light on these potential interactions. Currently, it is unknown whether newer PET tracers, like previous generations, bind to elements that are enriched in the LC (e.g., monoamine oxidases, neuromelanin), which would limit their use in selective imaging of tau in the LC. The need to develop and validate PET tracers also extends to other neurodegenerative disorders and neuropathology, such as PD and α-synuclein (Kotzbauer et al., 2017). Regardless, correlating pathological load with other measures of LC structure (contrast) and function (connectivity) could be useful for differentiating between disease-specific progressions.

Apart from pathological load, the NET PET radioligand 11C-MeNER can be used to estimate dysfunction in LC axon terminals and target regions. Several studies in PD cohorts have consistently reported lower 11C-MeNER binding in LC projection areas such as the thalamus, red nucleus, and motor cortex (Knudsen et al., 2018; Nahimi et al., 2018; Sommerauer et al., 2018a,b). However, reductions in 11C-MeNER binding within the LC proper and some other brain regions appear to be specific to PD patients with concomitant RBD (Sommerauer et al., 2018a). Interestingly, RBD patients without PD show similar decreases in LC contrast compared to those with PD, but appear relatively spared from terminal region abnormalities measured with 11C-MeNER PET (Knudsen et al., 2018). These results indicate complexities in LC dysfunction between two highly comorbid diseases, and suggest an interactive effect of RBD and PD that may be relevant for therapeutic intervention. Moreover, these studies indicate that NET PET imaging could help differentiate between different diseases and even sub-categories of disease (i.e., PD with/without RBD) using multiregional analysis. Whether these changes indicate outright axonal denervation and/or downregulation of NET has yet to be determined, which could be important for differentiating disease-specific progression and choice of treatment. Given the similarities between lower LC contrast in RBD and PD, it is perhaps unsurprising that contrast does not correlate well with 11C-MeNER binding in regions other than the thalamus (Sommerauer et al., 2018a), which receives some of the densest innervation from the LC (Jones and Moore, 1977; Jones and Yang, 1985; Morrison and Foote, 1986; Vogt et al., 2008). Sub-sectioning the LC along its various axes may reveal altered patterns of NET binding within the LC, although this will likely be difficult due to spatial resolution constraints. Only a single NET PET AD study has been conducted using (S,S)-18F-FMeNER-D2, and demonstrated lower binding in the LC and thalamus which correlated with Braak staging in post-mortem tissue (Gulyás et al., 2010). Clearly, the use of NET PET should be expanded to other neurodegenerative disorders, and classic studies should be replicated with new generation tracers. Similarly, adrenergic receptor distribution, which appears to be altered in disease-specific patterns (Xu et al., 2012), should also be investigated using PET. Abnormal signaling through adrenergic receptors can increase tau phosphorylation (Wang et al., 2013; Zhang et al., 2020), and disrupt neurotoxic compound clearance and increase inflammation through astrocytes (Leanza et al., 2018). Yet, it is unclear how expression levels of these receptors change throughout aging and during disease progression, which can be partially attributed to an α-adrenergic receptor subtype study bias and mixed results from tracer development studies (Shiue et al., 1998; Hume et al., 2000; Marthi et al., 2002; Lehto et al., 2015; Krzyczmonik et al., 2019). Finally, PET approaches utilizing longitudinal and cross-sectional designs would be useful for understanding the progression of dysfunction within the LC itself, in terminal regions, and, when combined with multimodal MRI, how these measurements relate to changes in LC integrity and connectivity.

## Animal Studies

Although in its infancy, emerging techniques have paved the way for both structural and functional LC neuroimaging in animal models of neurodegenerative disease that come with distinct advantages over human studies, including contribution of single risk gene variants and specific forms of pathology on disease progression, ability to assess longitudinal changes, and selective control of circuits in disease states using optogenetics and chemogenetics. Combinatorial deployment of these applications may be an especially powerful, but so far underutilized, approach to further investigate LC contributions to neurodegenerative disease. While new techniques have been developed for imaging the LC in humans, several obstacles have hindered progress in animals. The most common animals used to model neurodegenerative diseases are rodents, which due to their small brain size necessitate higher spatial resolution. Furthermore, rodents naturally lack neuromelanin, robbing investigators of a key tool (neuromelanin-sensitive MRI) for imaging the LC. Finally, faithful recapitulation of the range of neurodegenerative pathology (including early LC involvement) and behavioral manifestations akin to the human condition is rare in rodent models. Thus, we advocate for more LC imaging studies in rodents, as well as the advancement of non-human primate models of neurodegeneration, where natural aging processes more closely resemble that of humans.

### Structural MRI

As mentioned above, rodents lack endogenous neuromelanin. However, a recent study used a viral vector approach to overexpress human tyrosinase (which is involved in the production of melanin in the skin) in the substantia nigra of rats and mice, which led to age-dependent neuromelanin production (Carballo-Carbajal et al., 2019). After 2 months, hyperintensities consistent with neuromelanin production were evident in *ex vivo* T1-weighted MRI. If applicable to the LC, this method would allow neuromelanin-sensitive MRI of noradrenergic nuclei in rodent models of neurodegenerative disease. It is important to note that neuromelanin production also triggered age-dependent degeneration of the substantia nigra, but neuromelanin does not, by itself, appear to kill catecholaminergic neurons in humans (Zucca et al., 2017). Indeed, neuromelanin content in surviving LC neurons of AD patients is comparable to age-matched controls (Mann et al., 1980). Thus, while this viral vector approach provides an interesting PD-relevant model of catecholaminergic cell death, it may have limited face validity and broad applicability. Neuromelanin is hypothesized to induce neurotoxicity by binding heavy metals (Zucca et al., 2017), which warrants further investigation in this model and would clarify its application to human disease. It is possible that neuromelanin-dependent degeneration could be prevented or delayed by reducing the degree of tyrosinase overexpression with a weaker viral promoter or taking a transgenic/knockin approach. Researchers have also developed alternatives to segmenting the LC in rodent models without dependency on neuromelanin. Watanabe et al. (2019) were able to delineate the LC from other brainstem nuclei using a RF-spoiled 3D FLASH sequence, and detected changes in LC contrast in the triple transgenic APP/PS1/Ear2(−/−) mice compared to controls (APP/PS1 alone). This study suggests that high water proton density in cell bodies, rather than neuromelanin or the NE biosynthetic enzyme dopamine β-hydroxylase, is a source of LC contrast. It seems likely that multiple signals contribute to LC hyperintensities, which may each correspond to different properties of nuclei health. In fact, animal models are well suited to determine if and which measures of LC contrast correlate with different properties of nuclei health by combining imaging, histology, and LC-related perturbations (i.e., DSP-4 lesions, expression of human tyrosinase, etc.). Use of these imaging protocols in models of disease can further parse out effects of single risk gene variants and single pathologies on LC integrity.

While rodents offer superior genetic and viral strategies, they do not recapitulate the age-dependent deposition of amyloid and tau observed in humans (Van Dam and De Deyn, 2006). The reasons for this have not been fully delineated, but short life span, altered amyloid processing, and different isoforms/isoform ratios of tau are likely candidates. Some non-human primates develop amyloid deposits and even accumulate aberrant tau during the course of normal aging, much like humans (Li et al., 2019). Thus, non-human primates may help bridge the gap between preclinical animal models and humans by better recapitulating the process of normal aging. Additionally, since non-human primates produce neuromelanin in catecholaminergic nuclei (Scherer, 1939; Adler, 1942; Herrero et al., 1993; McCormack et al., 2004), similar to humans, the use of neuromelanin-sensitive MRI to investigate LC-related changes in these neurodegenerative models is possible. However, at this time, only a single study has investigated LC volume loss in MPTP-induced PD in marmosets using voxel-based morphometry at 7T (Hikishima et al., 2015). The paucity of non-human primate imaging studies is likely due to a number of factors including cost, underdeveloped models of neurodegenerative disease and a lack of genetic and opto/chemogenetic tools to selectively manipulate circuits (although these models and tools are now coming online; see Emborg, 2017; Galvan et al., 2018; Marmion and Kordower, 2018; Li et al., 2019). Regardless, current toxicity models, such as MPTP-induced PD and DSP-4 LC lesions, are able to capture compensatory changes in the LC (i.e., increased firing rate in response to cell death (Chiodo et al., 1983; Logue et al., 1985) that may be absent in models that include LC pathology but lack frank neuronal loss. Structural LC imaging in these toxicity models combined with post-mortem stereology could help disentangle the different sources of contrast, including the contributions of neuromelanin which is not possible in rodents. Development of transgenic non-human primates is also ongoing, and could be especially important for understanding genetic contributions to progression of endogenous neuropathology within the LC across aging and disease (Arnsten et al., 2019; Seita et al., 2020). Studies should also incorporate other imaging modalities in non-human primate models to better understand the effects of these manipulations on LC axon health and connectivity.

### Functional MRI

fMRI studies in animal models have enormous potential to contribute to our understanding of LC activity in normal and disease states, but are complicated by the aforementioned structural imaging caveats, specifically with regard to segmenting the nucleus. However, workarounds are possible, and animal models afford the opportunity to assess how circuit manipulations of the LC influence functional connectivity between downstream brain regions. Historically, electrical stimulation and pharmacology have been the manipulations of choice, but these interventions are not cell type-specific and cannot precisely control the rate and timing of stimulation. By combining various circuit-based techniques with MRI, such as optogenetics and chemogenetics, it is now possible to perturb specific brain regions, such as the LC, in a cell type-specific manner to interrogate their effects on brain-wide network activity and connectivity (Lee et al., 2010; Zerbi et al., 2019). Methods have also been developed to simultaneously perform MRI and electrophysiology (Shmuel and Leopold, 2008; Pan et al., 2010, 2013), which would be useful for investigating how baseline LC activity correlates with functional connectivity measures. Because these techniques are challenging to employ on their own, they have yet to be combined with animal models of disease. However, experiments combining fMRI with circuit-based manipulations or animal models have been carried out, and provide interesting insights into LC function during health and disease.

Mutations in the genes encoding amyloid precursor protein (APP) and presenilin-1 (PS1) cause AD in humans (Tang and Gershon, 2003). Interestingly, TgF344-AD rats harboring mutant human APP and PS1, unlike mice with the same transgenes, develop hyperphosphorylated tau in the LC prior to other vulnerable brain regions (Rorabaugh et al., 2017). Two fMRI studies have demonstrated that functional connectivity deficits in these rats are temporally linked to hyperphosphorylated tau deposition in the LC at an age when no other appreciable neuropathology is present (Muñoz-Moreno et al., 2018; Anckaerts et al., 2019). Longitudinal analysis revealed age-dependent deterioration of functional networks in parallel with worsening tau pathology in the LC, and the appearance of plaques and tangles in forebrain regions (Cohen et al., 2013; Rorabaugh et al., 2017), a pattern similar to that observed in human AD patients (Balachandar et al., 2015; Badhwar et al., 2017). Similarly, while multiple brain regions develop α-synuclein pathology in PINK1 knockout rat models of PD, the LC appears to be selectively vulnerable to frank neuronal loss (Grant et al., 2015; Cullen et al., 2018; Kelm-Nelson et al., 2018). fMRI again revealed that loss of TH immunoreactivity was temporally linked to functional connectivity abnormalities in PINK1 knockout rats (Cai et al., 2019). In one of the only studies we found that directly investigated functional connectivity of the LC in rodents, an increase in LC-ventral tegmental area connectivity was reported in PINK1 knockout rats that was associated with anxiety-like behaviors. This finding is similar to associations between LC-ventral tegmental area connectivity patterns and cognitive deficits over the course of normal aging in humans (Jacobs et al., 2018). Work such as this is crucial for detailing the neurobiological effects of single pathologies and disease-causing variants on functional connectivity, with initial studies hinting at early deleterious effects on the LC-NE system. Future studies that carefully characterize baseline functional connectivity patterns of the LC in rodents and non-human primates will be essential for determining the translatability of such work. fMRI of the LC in animal models would benefit from species-specific standardization and optimization of imaging parameters, including the creation of a histologically validated location map of the LC (similar to Keren et al., 2009).

A recent, landmark study in rodent LC neuroimaging utilized chemogenetics to stimulate the LC during fMRI in wild-type mice (Zerbi et al., 2019). Notable increases in brain-wide functional connectivity, including the salience, amygdalar, association, hippocampal, striato-motor, and default mode-like networks, suggest that LC activity can rapidly reconfigure brain state. Importantly, areas modulated by LC activation overlap with dysfunctional networks in rodent models of neurodegeneration (Grandjean et al., 2014; Muñoz-Moreno et al., 2018; Anckaerts et al., 2019; Cai et al., 2019) and human patients (Hacker et al., 2012; Luo et al., 2014; Balachandar et al., 2015; Badhwar et al., 2017). Chemogenetic Gq-DREADD stimulation appears to preferentially increase tonic LC firing rates (Armbruster et al., 2007; Vazey and Aston-Jones, 2014), suggesting that elevated tonic LC activity is associated with non-specific increases in brain-wide connectivity. Notably, the firing frequency and pattern elicited by chemogenetic activation is reminiscent of the LC under stress (Valentino and Foote, 1988; Curtis et al., 2012; McCall et al., 2015), indicating that functional connectivity changes may be specific to an anxiety-like state. Better temporal and patterned control is possible with optogenetics, and can be used to elucidate the relative contribution of tonic versus phasic firing patterns to functional connectivity. Moreover, Zerbi et al. (2019) provide evidence that the source of LC fMRI signal may be due to NE actions on adrenergic receptors through volume transmission/non-synaptic release from varicosities (Atzori et al., 2016; Mather et al., 2016), rather than via LC modulation of vasculature which is primarily vasoconstriction (Goadsby et al., 1985; Bekar et al., 2012). Due to emerging evidence of the LC’s modularity (Chandler et al., 2014; Uematsu et al., 2017), we also suggest the use of methods that are specific to particular LC projections. Because discrete groups of LC neurons appear to form ensembles (Totah et al., 2018; Noei et al., 2020), simultaneous LC recordings and fMRI could determine whether disease-specific pathologies disrupt intra-LC networks that may be associated with projection-specific deficits in functional connectivity.

### Positron Emission Tomography

Similar to humans, development of tracers for use in animal models of neurodegenerative disease has been complicated. Current microPET and autoradiography protocols appear capable of delineating NET-dense brain regions like the thalamus, cortex, and even the LC in rodents both *in* and *ex vivo* (Kirjavainen et al., 2018; López-Picón et al., 2019), though spatial resolution limits clear demarcation of LC boundaries. Extending NET microPET to other LC projection regions in these animal models of disease would allow longitudinal *in vivo* visualization of LC axons and terminals, and, when combined with immunohistochemistry, provide a better understanding of whether changes in retention represent downregulation of NET itself and/or axon terminal degeneration. Because hyperphosphorylated tau is detectable in the LC prior to any other brain region in both humans and some rodent models of AD (e.g., the TgF344-AD rat), developing an imaging protocol for LC tau microPET is also of interest. Unfortunately, the popular tau tracer 18F-AV-1451 demonstrates similar binding issues in animals as it does in humans. Rhesus monkeys, unlike other non-human primates, do not develop tau pathology during aging but demonstrate 18F-AV-1451 off-target signal (Hostetler et al., 2016), which, like humans, is most likely attributable to monoamine oxidase A binding. Even imaging the hippocampus in humans with 18F-AV-1451 is difficult, as the tracer demonstrates off-target binding to the adjacent choroid plexus (Lowe et al., 2016). Mice expressing mutant human P301L tau also appear to have issues with 18F-AV-1451 retention (Xia et al., 2013; Declercq et al., 2016). Other families of tau tracers show better results in rodents. Specifically, 11C-PBB3 in P301S and 18F-THK5117 in both P301S and biGT tau mouse models demonstrate retention in the brainstem and other areas (Maruyama et al., 2013; Brendel et al., 2016). Although tau, microPET imaging overlapped with immunohistochemical staining of tau deposits, future studies should utilize immunohistochemistry to determine the contributions of tau pathology in specific brainstem nuclei such as the LC to microPET signals, rather than treating the brainstem as a homogenous area. Additionally, the use of various tracers in different models of disease makes it difficult to compare studies (Saint-Aubert et al., 2017). Furthermore, the THK radiotracer family demonstrates high white matter binding in humans, which precludes visual analysis of PET images (Villemagne et al., 2014; Harada et al., 2015). These cross-species issues hamper translatability and highlight the pressing need to standardize imaging procedures, investigate isoform-specific tracer binding properties, and even limit the use of tracers to single diseases that are similar to others but show distinct pathology (Saint-Aubert et al., 2017). Supporting the latter idea is evidence that 11C-PBB3 appears to capture 4-repeat isoform tau specific to progressive supranuclear palsy and Pick’s disease, whereas 18F-AV-1451 may be better at capturing 3- and 4-repeat tau isoforms present in AD (Lowe et al., 2016; Sander et al., 2016; Ono et al., 2017).

Another informative use of microPET is to probe changes in excitatory and inhibitory balance throughout the brain, which is thought to be disrupted in various neurodegenerative disorders (Hynd et al., 2004; King et al., 2016). In the rTg4510 mouse tauopathy model, 11C-flumazenil (tracer for GABAA benzodiazepine receptors) binding was reduced beginning at 2 months of age, prior to frank neurodegeneration (Shimojo et al., 2020). Similar decreases in 11C-flumazenil binding are noted in early AD patients (Pascual et al., 2012). Although (E)-11C-ABP688 (tracer for mGluR5 receptors) binding failed to reveal a significant effect on excitatory synapses at this age, tau was evident in these neurons. Reductions in inhibitory innervation onto these tau-containing excitatory neurons led to network hyperexcitability prior to degeneration. Similarly, the LC develops hyperphosphorylated tau prior to other vulnerable brain regions in AD and does not degenerate until late stages of disease. Thus, tau-related decreases in inhibitory tone would be consistent with evidence of LC hyperactivity in prodromal and early AD (Weinshenker, 2018). A similar experiment in a model of synucleinopathy would be useful for evaluating the effects of α-synuclein on inhibitory and excitatory synapses.

## Conclusion and Considerations for Future Studies

The LC is among the earliest brain regions affected by neurodegenerative disorders, and appears susceptible to developing associated neuropathological hallmarks of disease. Development of these aggregates appears time-locked to prodromal symptoms of neurodegenerative disease which are indicative of LC dysfunction. Although prone to developing toxic aggregates, the LC appears largely spared from frank neuronal loss until mid to late stages of disease. Thus, mapping the progression of LC dysfunction in neurodegenerative disease in a non-invasive, *in vivo*, and longitudinal manner is of great interest and clinical utility.

In recent years, structural MRI of the LC has blossomed due to development of neuromelanin-sensitive techniques, and it has also become possible to coregister anatomical and functional scans to localize the LC and thus define functional connectivity of the LC at rest or during task engagement in health and disease. These early imaging results are largely consistent in reporting lower LC contrast and disrupted functional connectivity in disease, even though slightly different imaging parameters were used. Other structural imaging techniques, like DTI, have revealed axonal dysfunction in neurodegenerative disorders which has been supported by NET PET imaging. Unfortunately, DTI is unable to distinguish whether afferent or efferent LC projections are disrupted, whereas PET is unable to differentiate downregulation of NET versus outright axon terminal degeneration. Furthermore, although PET can also be used to measure pathological load within the LC, tracer issues largely limit its use as a biomarker for disease progress. Many of the early studies focused on the two most prevalent neurodegenerative disorders, AD and PD, but should be expanded to other diseases where LC dysfunction is thought to be involved (see Box 1 in Betts et al., 2019b). Using multimodal imaging to dissociate similar diseases may identify LC dysfunction that is disease-specific and help inform therapeutic interventions. Applying these imaging techniques across disease progression could contribute to disease staging in a minimally invasive and relatively inexpensive manner, although the full spectrum of LC-related dysfunction in disease needs to be further defined.

Moving forward, it will be necessary to determine if and how various LC imaging outcomes are related to one another. This inherently requires the refinement of PET tracers that track pathological load to reduce off-target binding. To investigate these relationships in other diseases (i.e., PD) or with respect to other LC-related elements (i.e., adrenergic receptors), new tracers will need to be developed, but have the potential to reveal new patterns of LC dysfunction. fMRI would also benefit from linking LC functional connectivity with LC contrast, pathological load, or NET density. It would be particularly interesting to determine whether patterned LC contrast correlated with functional connectivity, particularly based on innervation patterns. For example, the rostral and middle portions of the LC, which innervate the cortex and hippocampus, show lower contrast in AD, but it is unknown if this low signal intensity correlates with disrupted functional connectivity between the LC and these regions. Patterns of LC connectivity are also being related to tasks performed within or outside of the scanner. However, most of these have focused on memory tasks and should also consider other paradigms that could be influenced by LC activity, including novelty, arousal, attention, and stress. Post-mortem studies may assist in validating and determining the molecular and cellular basis of LC changes detected by fMRI and PET.

At this juncture, most LC imaging has been done in humans, leaving animal models of disease as an untapped resource for understanding the contribution of LC-NE dysfunction. There are many advantages to neuroimaging in animal models of neurodegenerative disease: (1) interrogation of single gene risk variants, (2) isolation of individual pathologies contributions to disease, (3) longitudinal assessment of changes throughout disease progression on a condensed time scale, and (4) selective manipulation of key brain regions and circuits using chemogenetics or optogenetics. Structural MRI of the LC is possible in animal models and could be particularly useful in parsing out the functional readouts of LC contrast (i.e., cell body versus dendritic field loss). fMRI in rodent models is also possible and so far mirrors results found in human neurodegenerative disease. Manipulation of the LC-NE system, and even selective circuits, during scanning can be accomplished with optogenetics and chemogenetics, and may be able to determine how tonic and phasic firing uniquely alter functional connectivity patterns. Use of optogenetics and chemogenetics could also identify circuits that are potential targets for therapeutic interventions at various stages of disease. However, baseline functional connectivity of the LC in rodents needs to be investigated to determine translatability to the human condition. MicroPET appears less well developed for many of the same reasons as human PET, but also due to spatial resolution constraints which make it difficult to fully delineate the LC. Spatial resolution could be remedied by performing *ex vivo* immunohistochemistry to localize neuropathological deposits and may provide a better understanding of whether NET PET readouts faithfully represent axon terminal integrity or are more aligned with NET protein downregulation. Comparatively, non-human primates have been utilized sparingly but have well developed toxicity models that mirror LC dysfunction seen in neurodegenerative disease. Furthermore, non-human primates could be particularly useful for investigating abnormalities in the process of normal aging and the relationship between neuromelanin accumulation and LC function.

An imperative consideration for the field going forward is the standardization of imaging procedures (which has been reviewed for MRI in Mandino et al., 2019). For example, the most commonly used anesthetic regimens for rodent MRI studies include isoflurane, dexmedetomidine, or a combination of the two. While this drug combination during resting-state fMRI recapitulates awake functional connectivity better than either drug alone (Paasonen et al., 2018), dexmedetomidine is an agonist of the α2-adrenergic inhibitory autoreceptor that dampens LC activity (Jorm and Stamford, 1993; Chiu et al., 1995; Zerbi et al., 2019; Reimann and Niendorf, 2020), making it incompatible for certain fMRI studies. Studies in awake animals are challenging but possible, meaning development of cross-species LC-influenced tasks would be useful for future studies. PET studies require similar investigation, as isoflurane increases tau phosphorylation, which could be exacerbated by mutant tau (Dong et al., 2012; Feng et al., 2016). Standardization should also be considered for human studies, and PET experiments have highlighted the need to standardize across species, where possible, to facilitate translatable results.

With recent developments in the imaging field, it is now possible to reliably measure multiple indices of LC health *in vivo* during aging and in disease. Early studies utilizing these methods have significantly advanced our knowledge of noradrenergic dysfunction in neurodegenerative disorders. While great strides have been made in human imaging, improvements can still be made in imaging LC axonal fiber integrity with MRI and neuropathological load using PET. Progress in these areas could assist in clinicians in diagnosing neurodegenerative diseases earlier and more accurately if combined with other biomarkers, and could lead to development of therapeutic agents targeting the noradrenergic system. Comparatively, few animal studies have focused on LC imaging, but represents a fertile ground for future research.

## Author Contributions

All authors contributed to the conception and revision of the manuscript, read and approved the final submission. MK wrote sections of the manuscript and created the figures.

## Conflict of Interest

The authors declare that the research was conducted in the absence of any commercial or financial relationships that could be construed as a potential conflict of interest.
